# Quantification of pharmacokinetic profiles of a recombinant canine PD-1 fusion protein by validated sandwich ELISA method

**DOI:** 10.3389/fvets.2022.951176

**Published:** 2022-08-03

**Authors:** Jicheng Qiu, Yuxin Yang, Jingyuan Kong, Yuying Cao, Yu Liu, Haoshu Luo, Xingyuan Cao

**Affiliations:** ^1^College of Veterinary Medicine, China Agricultural University, Beijing, China; ^2^Laboratory of Quality and Safety Risk Assessment for Animal Products on Chemical Hazards (Beijing), Ministry of Agriculture and Rural Affairs, Beijing, China; ^3^College of Biological Sciences, China Agricultural University, Beijing, China; ^4^Beijing VJTBio Co., LTD., Beijing, China; ^5^Laboratory of Detection for Veterinary Drug Residues and Illegal Additives, Ministry of Agriculture and Rural Affairs, Beijing, China

**Keywords:** canine tumor, PD-1/PD-L1, immunotherapy, ELISA, pharmacokinetics

## Abstract

Tumors are becoming a serious threat to the quality of life of human and dogs. Studies have shown that tumors have caused more than half of the deaths in older dogs. Similar to human, dogs will develop various and highly heterogeneous tumors, but there are currently no viable therapies for them. In human, immunotherapy has been used widely and considered as an effective treatment for tumors by immune checkpoint targets, which are also expressed on canine tumors, suggesting that immunotherapy may be a potential treatment for canine tumors. In this work, we developed a sandwich ELISA method to detect the concentration of recombinant canine PD-1 fusion protein in canine serum and investigated pharmacokinetics in canines after intravenous infusion administration. After being validated, the ELISA method showed an excellent linear relationship in 25.00–3,200.00 ng/ml in serum, and the *R*^2^ was more than 0.99 with four-parameter fitting. The precision and accuracy of intra-assay and inter-assay at the five different concentrations met the requirements of quantitative analysis. At the same time, no hook effect was observed at the concentration above ULOQ, and the stability was good under different predicted conditions with accuracy > 80%. The pharmacokinetic study in dogs has shown that the recombinant canine PD-1 fusion protein exhibited a typical biphasic PK profile after intravenous infusion administration, and the linear pharmacokinetic properties were observed between 1.00 and 12.00 mg/kg. Meanwhile, the T_1/2_ after intravenous infusion administration with non-compartmental analysis was about 5.79 days.

## Introduction

With rapid development in medicine and diagnosis for companion animals, infections are no longer the major cause of mortality in humans and companion animals because of the use of antibiotics, but the prevalence of malignancies is increasing (1, 2). Tumors have been a major threat to animals' life. Tumors affect ~50% of older dogs, and ~25% of dogs eventually die from tumors (3, 4). Tumors occur most frequently in the skin and soft tissues, with an incidence of 1,437 per 100,000 dogs per year (5). They were localized in the skin and soft tissues, breasts, perineal region, and oral cavity of male dogs and in the skin and soft tissues, breasts, vagina, and perianal region of female dogs. Moreover, the types of tumors in canines, as in human, are complicated and diverse. Adenomas, mast cell tumors, skeletal tumors, and melanomas are the most prevalent tumors in canines (5–8), and this makes the canine a perfect model for human tumor therapy.

For tumors in canines, surgery is still the most common treatment in the clinic. It seems to be an effective solution for solid tumors with significant margins, but it is difficult to achieve an excellent result in some malignant and metastatic tumors, such as melanoma (9–11). On the contrary, radiotherapy seems to be another option for tumors in canines, but it seems to be suitable only for tumors of small size (12). In addition, some chemotherapeutic agents, such as doxorubicin, carboplatin, and cisplatin, have been used to treat tumors in canines, but they usually lead to a variety of side effects that may affect the quality of life. Therefore, noncytotoxic anticancer drugs, including tyrosine kinase inhibitors (TKs) (13–16), therapeutic proteins, and vaccines (17–19), appear to be the most promising treatments due to their targeted effects and low toxicity.

Immunotherapy has gained attention due to the potential of immune checkpoint inhibitors in cancer treatment, and programmed death-1/programmed death ligand-1 (PD-1/PD-L1) is the important immune checkpoint for cancer treatment, which has been evidenced in a variety of malignant tumors, such as non-small cell lung cancer and melanoma. PD-L1 can be overexpressed on tumor cells and tumor-infiltrating immune cells, which binds PD-1 expressed on activated T cells to suppress the activation of T cells and induce T-cell exhaustion, resulting in the immune evasion of cancer cells. Immune checkpoint inhibitors, such as monoclonal antibodies, and fusion proteins can block the binding of immune checkpoints by targeting PD-1 or PD-L1 to reactive T cells to eliminate tumor cells. Like in human, PD-L1 is also overexpressed on various tumor cells in dogs, such as oral malignant melanoma, hemangiosarcoma, and osteosarcoma, implying that it's also a promising approach for canine tumors through immune checkpoint inhibitors (20, 21). Some therapeutic proteins, such as PD-1 monoclonal antibody (22) and canine PD-L1 chimeric monoclonal antibody (21, 23), have been shown to be effective for canine tumors *via* the PD-1/PD-L1 pathway. However, the pharmacokinetics of therapeutic proteins for canine tumors has not yet been reported.

Unlike small-molecule drugs, the pharmacokinetics of therapeutic proteins is characterized by slow absorption, low volume of distribution, slow elimination, and long elimination half-life due to molecular size and hydrophilicity (24). Therefore, studying the pharmacokinetics of therapeutic proteins is important for the development of anticancer drugs in both dogs and human. Recombinant canine PD-1 fusion protein is a new anticancer therapeutic protein for the PD-1/PD-L1 pathway, which is composed by linking the Fc fragment of canine IgG and the extracellular region of PD-1. In this study, an enzyme-linked immunosorbent assay (ELISA) method was developed and validated to study the pharmacokinetics of recombinant canine PD-1 fusion protein in canines after intravenous infusion administration.

## Materials and methods

### Reagents

Recombinant canine PD-1 fusion protein (10 mg purity powder/bottle), anti-recombinant canine PD-1 fusion protein antibody (coating antibody), and hIgG/HRP (detecting antibody) were provided by Beijing Weijiexin Medicine Technology Co., Ltd. (Beijing, China). Bovine serum albumin (BSA), coating solution, 3,3',5,5'-tetramethylbenzidine (TMB), and phosphate-buffered saline with 0.05% Tween-20 (PBST) were purchased from Solarbio (Beijing, China). Dulbecco's phosphate-buffered saline (DPBS) was purchased from Corning (New York, USA). LowCross-Buffer was purchased from Candor Bioscience (Wangen, Germany).

### ELISA method development

To develop a highly sensitive and specific ELISA method, the coating antibody was diluted with coating buffer in the range of 0.20–5.00 μg/ml, and the detecting antibody was diluted with DPBS in the range of 0.10–0.50 μg/ml. Meanwhile, the coating condition was selected as 37°C for 2 h or 4°C overnight, and the blocking time was compared ranging from 30, 60, 90 to 120 min in 5% BSA solution. In addition, the incubation time of the detecting antibody was optimized from 30, 60 to 90 min.

### Recombinant canine PD-1 fusion protein ELISA assay

A volume of 1 μg/ml anti-recombinant fusion canine PD-1 protein antibody diluted with coating solution was added into the Corning Costar™ 96-well high-binding microtiter plate (100 μl/well) and then coated overnight at 4°C. After being washed four times with 300 μl/well of PBST, the wells were blocked with 300 μl of 5% (w/v) BSA diluted with DPBS for 2 h at 37°C. After being washed four times with PBST, the recombinant canine PD-1 fusion protein samples were diluted by 1:4 in LowCross-Buffer, and 100 μl of diluted samples were added to the wells and incubated for 1 h at 37°C. Following incubation, the plate was washed four times with PBST, and the detecting antibody was diluted to 0.125 μg/ml in DPBS, and 100 μl was then added into the wells, which were then incubated for 1 h at 37°C. At the end of the incubation period, after being washed four times with PBST, adding 100 μl TMB to each well, the plate was then incubated in the dark at 37°C for up to 30 min, and the reaction was stopped by adding 50 μl/well ELISA stop solution. The subsequent absorbance was quantified by the measurement at 450 nm using a 96-well plate reader (Multiskan FC, Thermo), and the results were analyzed using software (Skanlt RE 6.1.1, Thermo).

### ELISA method validation

A comprehensive validation of the ELISA method was performed according to the ICH M10 on bioanalytical method validation (25) and the FDA bioanalytical method validation guidance for industry (26), which are generally accepted rules for bioanalytical method validation. The linearity, accuracy, precision, dilution linearity, hook effect, parallelism, and stability were evaluated in detail.

#### Linearity and calibration curve

The linearity of the ELISA method was determined by the replicate analysis of six complete standard curves on separate days. The standard of the recombinant canine PD-1 fusion protein was 2-fold diluted with blank canine serum ranging from 12.50 to 6,400.00 ng/ml to measure the linearity of the ELISA method. The standard curve was analyzed by plotting the absorbance against the logarithm of each concentration and fitted in a four-parameter logistic equation using Origin version 2021 (OriginLab, Northampton, MA, USA). The calibration curve was accepted if the rollback concentrations for at least 6 calibration standards were comprised within 20% of the theoretical concentration.

#### The accuracy and precision

The accuracy and precision were evaluated by testing the quality control (QC) samples containing five different amounts of recombinant canine PD-1 fusion protein at standard levels of 25.00, 60.00, 250.00, 500.00, and 1,000.00 ng/ml in blank canine serum on separate days. Accuracy was determined as the difference (% bias) between rollback concentration and theoretical concentration, and precision was shown as the coefficient of variation (CV%). For within-day and between-day precision and accuracy, the acceptance criteria included a %bias of no more than 20% deviation from the theoretical value and CV% ≤ 80%. The lower and upper limits of quantification (LLOQ and ULOQ) were quantified with precision and accuracy ≥ 75%. The total error in a test result is attributed to imprecision (%CV) and inaccuracy (%Bias), which should not exceed 30% (40% at LLOQ and ULOQ).

#### Dilution linearity and hook effect

Five repeats of 12,800.00 ng/ml (DQC1) and 25,600.00 ng/ml (DQC2) were prepared for hook effect evaluation. Meanwhile, the dilution linear samples were prepared by diluting three dilution factors, namely, 10, 50, and 100, from DQC1 or DQC2 to concentrations of 1,280.00 ng/ml (DQC3), 512.00 ng/ml (DQC4), and 128.00 ng/ml (DQC5) individually. The rollback concentration for each dilution should be within ±20% of the theoretical concentration after correction for dilution, and the precision of the final concentrations across all the dilutions should not exceed 20%.

#### Parallelism

To evaluate parallelism, the blood samples over ULOQ were diluted in drug-free serum by three different diluted factors (i.e., 1:50, 1:80, and 1:100) to measure the parallelism. The measured concentration should be included in the calibration range and should deviate from the theoretical value by no more than 20%.

#### Stability

To ensure stability during sample preparation, processing, and analysis, as well as the storage conditions, the sample was assessed under different conditions, such as stored at 4°C for 4 h, room temperature (RT) for 4 h and 1 day, three cycles of freezing and thawing, and stored at −20°C for 1 month at two different concentrations (60.00 and 2,400.00 ng/ml). The mean concentration at each level should be within ±20% of the theoretical concentration.

### Pharmacokinetics experimental design

In total, 18 healthy adult Beagles (half male and female), aged 14–24 months, weighted from 8 to 13 kg, and were obtained from Beijing Yuandaxinghuo Medicine Technology Co., Ltd. (Beijing, China), were chosen to study the pharmacokinetics of recombinant canine PD-1 fusion protein and were randomly divided into 3 groups. Before the start of the study, the blank serum was collected as the blank control sample, and the three groups of animals were administrated with 50 ml saline by intravenous infusion pump at the dose of 1.00 mg/kg (group L), 4.00 mg/kg (group M), and 12.00 mg/kg (group H), respectively, at the speed of 40 ml/h. The blood samples (~3 ml) were collected from veins of the forelimbs into a coagulation tube at the following time points: 0.083 h, 0.25 h, 0.5 h, 1 h, 2 h, 4 h, 8 h, 24 h (D1), 48 h (D2), 96 h (D4), 336 h (D14), and 504 h (D21). After being stored at 4°C for 40 min, the serum was separated by centrifugation at 4,000 rpm/min for 10 min and stored at −80°C until analysis.

### Statistical analysis

Non-compartmental analysis (NCA) using the WinNonlin^®^ 8.3.4 pharmacokinetic software (Pharsight Corporation, California, USA) was performed to determine the PK parameters of recombinant canine PD-1 fusion protein following a single intravenous infusion administration. The main pharmacokinetic parameters were calculated, including the area under the serum concentration-time curve from 0 to the last point of the measured concentration (AUC_last_), clearance (Cl), the maximum serum concentration (C_max_), elimination half-life (T_1/2_), and mean residence time (MRT_last_). The pharmacokinetic data were presented as mean ± standard deviation (SD).

All precision and accuracy data were calculated as follows:

Accuracy (% bias) = (Derived concentration/Actual concentration) 100.

Precision (%CV) = (Standard deviation of the replicates/Mean derived concentration) 100.

Total Error (TE) = |% bias |+|%CV|.

## Results

### ELISA development and optimization

**Figure 1:**
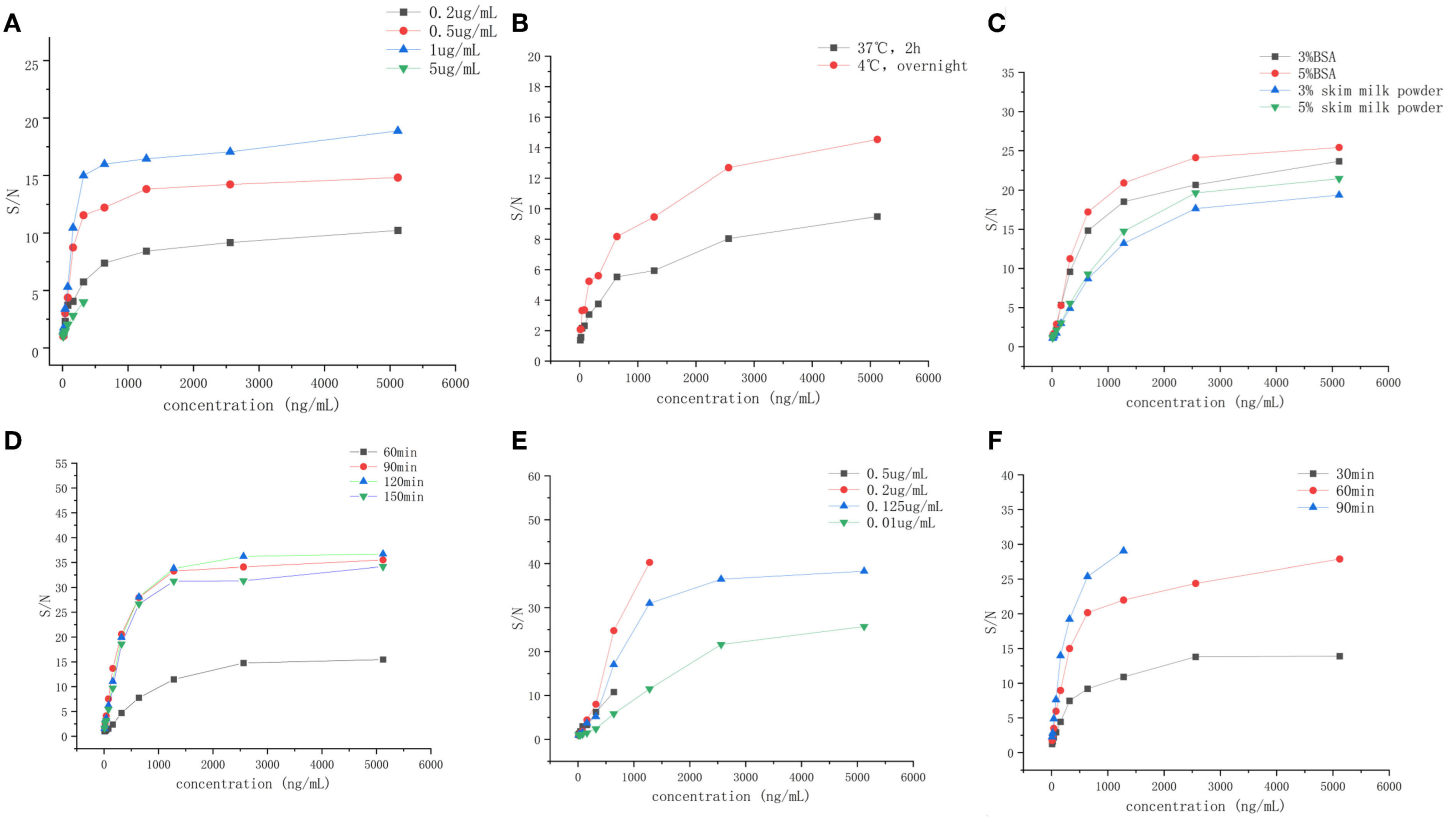
The establishment and optimization ELISA method of the recombinant canine PD-1 fusion protein. **(A)** The concentration of capture antibody (0.2, 0.5, 1, and 5 μg/ml. **(B)** The coating condition (under 37°C for 2 h, under 4°C overnight). **(C)** The blocking solution (3% BSA, 5% BSA, 3% skim milk powder, and 5% skim milk powder). **(D)** The blocking time (60, 90, 120, and 150 min). **(E)** The concentration of detection antibody (0.5, 0.2, 0.125, and 0.1 μg/ml). **(F)** Incubation time of detection antibody (30, 60, and 90 min).

After optimizing six different factors that could affect the sensitivity and detection range of the ELISA method, the ELISA detection procedure was carried out by applying 1 μg/ml of coating antibody coated on a 96-well plate overnight at 4°C. Then, the wells were blocked with 300 μl of 5% BSA for 2 h at 37°C. Finally, 100 μl of detecting antibody solution with 0.125 μg/ml concentration was added to the wells and incubated for 1 h at 37°C. The results are shown in Figure 1.

### ELISA validation

#### Linearity and calibration curve

**Figure 2:**
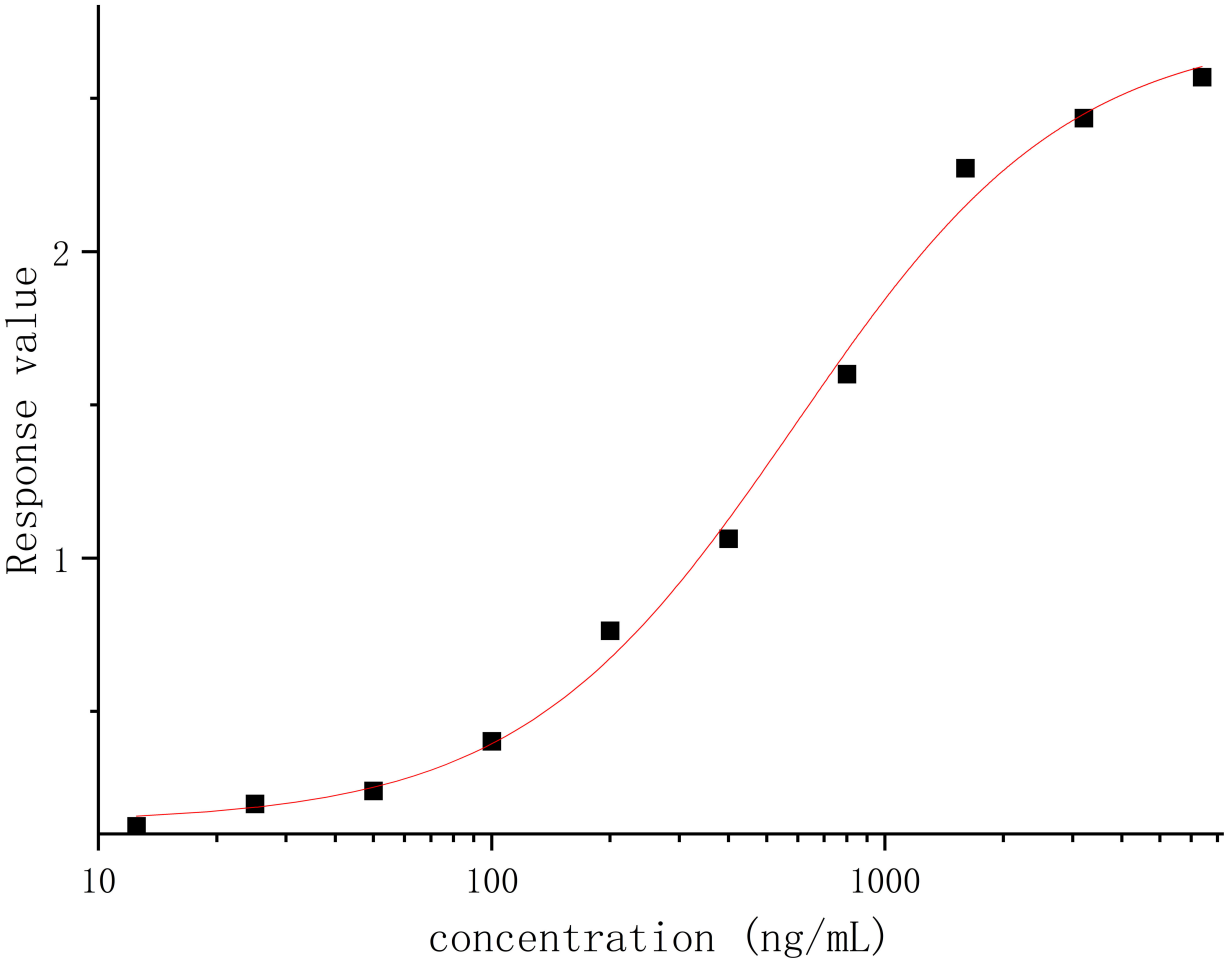
The response (OD) vs. recombinant canine PD-1 fusion protein concentration was analyzed by the four-parameter fitting using the Origin 2021 software.

The linearity was analyzed using eight calibration standards ranging from 25.00 to 3,200.00 ng/ml, and samples with concentrations of 6,400.00 and 12.50 ng/ml were set as anchor points to assist in establishing the standard curves, but they were out of the curve range. The response (OD) vs. the concentration of recombinant canine PD-1 fusion protein was analyzed by the four-parameter fitting using the Origin 2021 software. The correlation coefficient is >0.99 (Figure 2), indicating a good linear correlation in the range of 25.00–3,200.00 ng/ml. The rollback concentration of recombinant canine PD-1 fusion protein is shown in Table 1. At the same time, the accuracy of each concentration in the calibration standards was also analyzed and is shown in Table 1.

**Table 1:** The back-calculated concentrations and accuracy of the recombinant canine PD-1 fusion protein on calibration curve from 6 independent experiments.

**Theoretical concentration (ng/mL)**	**25.00**	**50.00**	**100.00.00**	**200.00**	**400.00**	**800.00**	**1,600.00**	**3,200.00**
Donor 1	22.83	57.58	97.89	203.58	404.84	748.53	1,774.10	2,988.54
% bias	−8.70	15.16	−2.11	1.79	1.21	−6.43	10.88	−6.61
Donor 2	23.42	52.09	106.13	194.16	395.32	829.60	1,492.48	3,592.78
% bias	−6.33	4.18	6.13	−2.92	−1.17	3.70	−6.72	12.27
Donor 3	23.26	52.04	112.52	181.57	410.75	812.98	1,512.53	3,549.94
% bias	−6.98	4.08	12.52	−9.22	2.69	1.62	−5.47	10.94
Donor 4	25.67	51.92	102.68	190.03	417.08	795.01	1,488.29	3,883.92
% bias	−7.40	7.53	0.19	−8.56	2.04	8.71	−2.17	−24.04
Donor 5	25.67	51.92	102.68	190.03	417.08	795.01	1,488.29	3,883.92
% bias	2.67	3.83	2.68	−4.99	4.27	−0.62	−6.98	21.37
Donor 6	24.40	56.46	96.87	188.77	425.62	800.73	1,453.01	3,697.62
% bias	−2.38	12.93	−3.13	−5.61	6.41	0.09	−9.19	15.55
Average back-calculated	24.14	53.78	102.73	189.72	415.66	795.99	1,529.40	3,615.02
%CV	4.73	5.09	5.13	4.08	3.31	3.42	7.14	9.54
% bias	−3.44	7.56	2.73	−5.14	3.92	−0.50	−4.41	12.97

#### Within-day and between-day precision and accuracy

Five QC samples at the levels of 25.00, 60.00, 250.00, 500.00, and 1,000.00 ng/ml in blank beagle serum were analyzed to measure the precision and accuracy of the ELISA method, and the results are shown in Table 2. Each level has three repeats in an intra-assay run and six times on different days in an inter-assay run. The intra-assay run %bias and %CV were in the range of 22.95–18.79% and 2.23–23.50%, respectively. The inter-assay run %bias and %CV ranged from 1.71 to 5.91% and 10.52 to 20.95%, respectively. The total error ranged from 8.99 to 32.27%.

**Table 2:** Evaluation of precision and accuracy of the recombinant canine PD-1 fusion protein determination in drug-free plasma.

**Donor**	**Actual spike (ng/mL)**	**25.00**	**60.00**	**250.00**	**2,400.00**	**3,200.00**
1	Average back-calculated (*n* = 3)	29.39	71.28	296.01	2,850.93	3,701.37
	Precision (%CV)	4.37	2.72	3.64	3.70	16.60
	Accuracy (%bias)	17.54	18.79	18.41	18.79	15.67
	TE (%)	21.91	21.51	22.05	22.49	32.27
2	Average back-calculated (*n* = 3)	29.85	68.08	258.97	2,652.29	3,619.00
	Precision (%CV)	5.19	3.39	5.40	2.44	8.64
	Accuracy (%bias)	19.39	13.46	3.59	10.51	13.09
	TE (%)	24.58	16.85	8.99	12.96	21.73
3	Average back-calculated (*n* = 3)	22.49	54.77	245.68	2,686.97	3,099.55
	Precision (%CV)	8.78	3.18	9.72	17.27	23.50
	Accuracy (%bias)	−10.05	−8.72	−1.73	11.96	−3.14
	TE (%)	18.83	11.80	11.45	29.23	26.64
4	Average back-calculated (*n* = 3)	22.53	59.10	283.90	2,403.36	3,016.06
	Precision (%CV)	15.57	2.99	11.66	13.32	15.63
	Accuracy (%bias)	−9.88	−1.50	13.56	0.14	−5.75
	TE (%)	25.45	4.49	25.22	25.22	21.38
5	Average back-calculated (*n* = 3)	24.79	60.75	229.42	2,129.26	2,465.68
	Precision (%CV)	14.91	6.00	2.29	3.52	3.39
	Accuracy (%bias)	−0.84	1.24	−8.23	−11.28	−22.95
	TE (%)	15.75	7.25	10.52	14.80	26.34
6	Average back-calculated (*n* = 3)	22.98	60.54	274.66	2,281.58	2,803.21
	Precision (%CV)	3.38	6.86	5.26	5.06	9.40
	Accuracy (%bias)	−8.10	0.89	9.86	−4.93	−12.40
	TE (%)	11.47	7.75	15.12	9.99	21.80
Inter-assay run	Average back-calculated (*n* = 18)	25.34	62.42	264.77	2,451.65	3,145.23
	Precision (%CV)	15.77	10.52	11.47	11.32	19.23
	Accuracy (%bias)	1.35	4.03	5.91	2.15	−1.71
	TE (%)	17.12	14.55	17.38	13.47	20.95

#### Dilution linearity and hook effect

**Figure 3:**
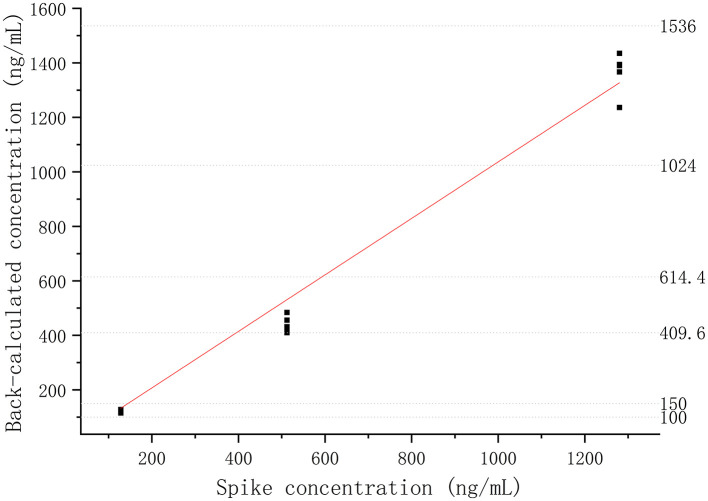
Dilutional linearity of the recombinant canine PD-1 fusion protein in drug-free plasma assessed by precision and accuracy (*R*^2^ > 0.99).

The absorbances of DQC1 and DQC2 were higher than the absorbance of ULOQ (3,200.00 ng/ml), indicating that there was no hook effect. The %CV of dilution samples was <6.16%, and the %bias was <14.08%, which means that the sample above the ULOQ could be quantified accurately and precisely after being diluted 10, 50, or 100 times, as shown in Figure 3.

#### Parallelism

As shown in Table 3, the mean values of the back-calculated concentration were 9,398.56, 10,360.70, and 10,812.01 ng/ml with a %CV of 4.79%, which confirmed that there was no significant deviation in incurred samples.

**Table 3:** Evaluation of parallelism of the recombinant canine PD-1 fusion protein determination in drug-free plasma with 3 different dilutions.

**Dilution factor**	**Back-calculated concentration**			**Mean (ng/mL)**	**Intra %CV**	**Inter %CV**
	**(ng/mL)**					
50	10,284.97	9,582.26	9,214.85	9,398.56	5.79	4.79
80	10,046.47	10,004.67	10,716.72	10,360.70	3.86	
100	10,046.47	10,004.67	10,716.72	10,812.01	3.35	

#### Stability

**Figure 4:**
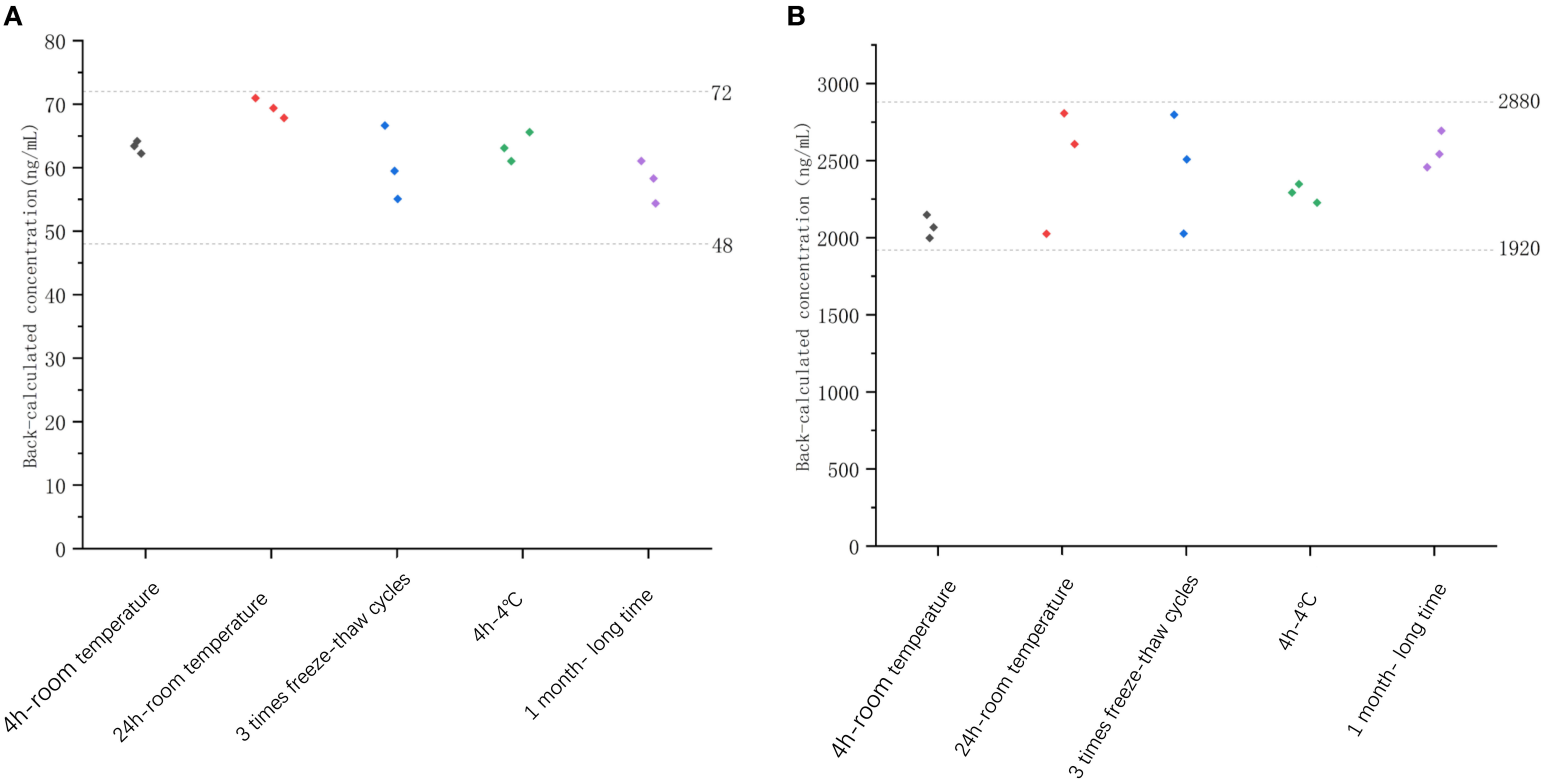
The stability evaluation of recombinant canine PD-1 fusion protein determination under different conditions with low QC concentration (60 ng/ml, **A**) and high QC concentration (2,400 ng/ml, **B**).

The stability of freeze–thaw was measured after three times of storing spiked samples at −20°C and thawing at room temperature. After 1 month of storage at −20°C, the long-term stability of recombinant canine PD-1 fusion protein was determined. As shown in Figure 4, the %bias of stability samples was <20% under these different conditions. Therefore, the samples were stable during storage and preparation.

### Pharmacokinetics in clinical trials subjects

**Figure 5:**
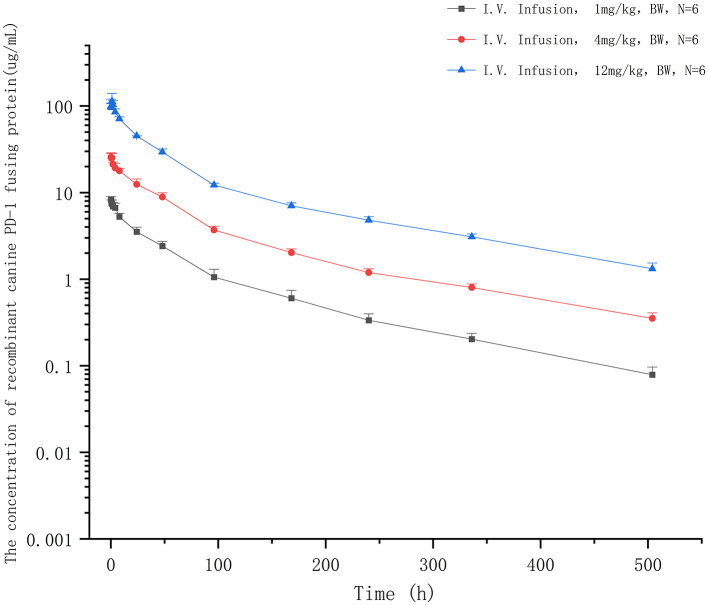
The concentration–time profiles of recombinant canine PD-1 fusion protein after a single intravenous administration of 1, 4, and 12 mg/kg in canine serum.

**Figure 6:**
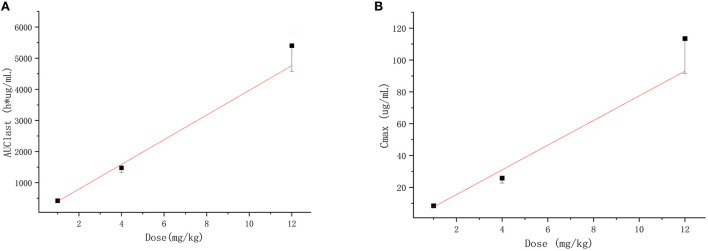
Dose-proportionality assessment of recombinant canine PD-1 fusion protein pharmacokinetics in healthy canine. **(A)** Mean ± SD for AUClast vs dose for subjects in each cohort with the linear regression line defined by *f* (*x*) = 396.833x, *r*^2^ = 0.993. **(B)** Mean ± SD for *C*_max_ vs. dose for subjects in each cohort with the linear regression line defined by *f* (*x*) = 7.755x, *r*^2^= 0.982.

The extensively validated ELISA method was successfully applied to determine the concentrations of the recombinant canine PD-1 fusion protein in the serum of canine after a single intravenous infusion administration of 1.00, 3.00, and 12.00 mg/kg. The mean plasma concentration–time profiles are shown in Figure 5. The concentrations of the recombinant canine PD-1 fusion protein in canine serum exhibited a typical biphasic PK profile with a relatively rapid distribution phase and a relatively slow elimination phase. The main pharmacokinetic parameters calculated using the WinNonlin software by the non-compartmental model are presented in Table 4. The T_1/2_ and clearance of recombinant canine PD-1 fusion protein appeared to be independent of the dose administered, and the C_max_ and AUC_last_ values increased linearly along with the dose, as shown in Figure 6. Therefore, recombinant canine PD-1 fusion protein exhibited linear pharmacokinetics over the dose range of 1.00–12.00 mg/kg.

**Table 4:** Summary of pharmacokinetic parameters (mean ± SD) for the recombinant canine PD-1 fusion protein in canines following IV infusion administrations.

**Parameter**	**L group (1 mg/kg)**	**M group (4 mg/kg)**	**H group (12 mg/kg)**
AUC_last_ (h*ug/mL)	420.417 ± 61.394	1,479.347 ± 156.632	5,400.691 ± 821.788
MRT_last_ (h)	92.069 ± 3.528	97.091 ± 2.272	97.357 ± 2.151
AUCINF_obs (h*ug/mL)	435.033 ± 63.587	1,555.901 ± 171.045	5,672.244 ± 820.828
λ_z_ (1/h)	0.006 ± 0.000	0.005 ± 0.000	0.005 ± 0.000
T_1/2_ (hr)	126.702 ± 18.893	149.044 ± 8.341	142.852 ± 9.630
C_max_ (ug/ml)	8.428 ± 0.655	25.853 ± 3.150	113.522 ± 21.864
Cl_obs (mL/h/kg)	2.339 ± 0.335	1.949 ± 0.229	2.147 ± 0.258
Vss_obs (mL/kg)	262.042 ± 36.633	248.359 ± 23.540	274.078 ± 42.697

## Discussion

The ELISA method has been widely used in medicine and food safety due to its high specificity and high sensitivity (27). This is the first study to demonstrate the pharmacokinetics of anti-tumor therapeutic proteins in canines with a validated ELISA method. To meet the requirements for quantitative analysis, it has been fully verified according to the guidelines launched by FDA and ICH. The standard curve is analyzed with the four-parametric fitting. It is generally S-shaped after log transformation and has an upper and lower platform, and the quantitative range should be in the middle section of the S curve (28, 29). The absorbance values varied significantly at the same concentration with different concentrations of the capture antibody and the detection antibody, which would affect the range and slope of the S-curve and change the quantitative range. Therefore, the concentration of the capture antibody and detection antibody was optimized first. The results show that the quantitative range was favorable when the detection antibody was diluted to 0.5 and to 0.125 μg/ml, and the quantitative range was between 25.00 ng/ml (LLOQ) and 3,200.00 ng/ml (ULOQ), and the precision and accuracy of each concentration on the standard curve were in accordance with the standard. In addition, the accuracy and precision were validated by more different concentrations, replicates, and batches than the validation of the UPLC-MS/MS method (25, 26), and both the precision and accuracy conform to the requirements. The hook effect will cause false-negative at high drug concentrations (30, 31), which was detected by adding the recombinant protein concentration over ULOQ. The absorbance values of hook effect samples are higher than that of ULOQ, which means that no hook effect was detected in the established ELISA method. In conclusion, a validated ELISA method was established to quantify the concentration of recombinant canine PD-1 fusion protein in serum.

Various components of the matrix may affect antigen-antibody binding, such as the presence of endogenous antibodies, phospholipids, hemoglobin, carbohydrates, and endogenous metabolites (bilirubin), which may lead to false-positive or false-negative results (32, 33). In this study, a visible decrease in absorbance values was observed when the concentration of recombinant canine PD-1 fusion protein in canine serum increased, suggesting there is a matrix effect in the ELISA method of recombinant protein in canine serum. Dilution with buffer solutions or water has been proven to be an effective means to eliminate matrix effects (34–36). At the same time, adding proteins such as BSA and ovalbumin (OVA) to the solution can also reduce the matrix effect, but nonspecific adsorption and an increase in background absorption should be noted due to the cross-reaction of proteins (37). In this study, DPBS, PBST, DPBS with 1%BSA, DPBS with 3% BSA, and LowCross-Buffer were used as dilution solutions to reduce matrix effects. With dilution in these solutions, the absorbance in the serum was the same as that in DPBS, indicating that the suppression of matrix interference was eliminated effectively. When serum samples were collected, varying degrees of hemolysis often occur, which may result in the release of a large amount of cellular content that interferes with antigen-antibody binding (38, 39). In this study, both hemolyzed and non-hemolyzed samples were used to analyze the effect of hemolysis on the ELISA method based on a 1:4 dilution with LowCross-Buffer, and no significant difference was observed between the two matrices.

Compared with small-molecule drugs, therapeutic proteins have special pharmacokinetic properties, such as lower distribution, low clearance, and degradation, by hydrolases instead of CYP450 enzymes (40, 41). At the same time, pharmacokinetics is influenced by many other factors, such as target-mediated drug disposition (TMDD) and anti-drug antibodies (ADA), which would lead to nonlinear pharmacokinetics (42, 43). Similar to most typical therapeutic proteins (44–46), recombinant canine PD-1 fusion protein demonstrated a two-phase pharmacokinetic profile typical of therapeutic protein, with a rapid tissue distribution phase followed by a slower elimination phase. The C_max_ and AUC_last_ values increased linearly along with the dose, which means that the linear pharmacokinetics was observed between 1.00 and 12.00 mg/kg administered. The linear pharmacokinetics was also obtained for other therapeutic proteins in healthy animals following a single dose (47, 48). PD-L1 is not expressed on cells in healthy animals, and there was no characteristic bend in the recombinant canine PD-1 fusion protein pharmacokinetics curve suggestive of TMDD. Meanwhile, after a single dose, the animals do not produce anti-canine PD-1 fusion protein antibodies, so the recombinant canine PD-1 fusion protein shows linear pharmacokinetics in canines after intravenous administration. The volume of distribution at steady state (V_ss_) was 0.26 L/kg, indicating a small volume of distribution similar to that with limited distribution beyond extracellular space, which provides sufficient availability for the drug binding PD-L1. Recombinant canine PD-1 fusion protein has a low clearance (2.145 ml/h/kg) and a long residence time (MRT_last_ = 95.506 h) after intravenous administration, suggesting that it exists for a long time. Simultaneously, the average T_1/2_ value was 139 h (5.79 days), indicating a low clearance in canines. But it may be higher than the actual half-life after multiple doses of administration in the clinic because of TMDD and ADA.

## Conclusion

This is the first study to report the pharmacokinetics of antitumor therapeutic protein agents in canines *in vivo* using a fully validated ELISA method after intravenous infusion administration. A novel and validated ELISA method was developed and optimized to determine the concentrations of the recombinant canine PD-1 fusion protein in canine serum and was successfully applied to study the pharmacokinetics of recombinant canine PD-1 fusion protein in canines.

## Data availability statement

The original contributions presented in the study are included in the article/supplementary material, further inquiries can be directed to the corresponding author.

## Ethics statement

The animal study was reviewed and approved by GCP Laboratory Animal Ethics Committee of China Agricultural University.

## Author contributions

XC conceived and designed the experiments. HL done antibody synthesis and expression. JQ, YY, YC, and YL performed the experiments. JQ and JK analyzed the data. The manuscript was discussed and reviewed by all authors. All authors contributed to the article and approved the submitted version.

## Funding

This study was financially supported by the National Key Research and Development Program (No. 2018YFD0500301).

## Conflict of interest

Author HL was employed by Beijing VJTBio Co., LTD. The remaining authors declare that the research was conducted in the absence of any commercial or financial relationships that could be construed as a potential conflict of interest.

## Publisher's note

All claims expressed in this article are solely those of the authors and do not necessarily represent those of their affiliated organizations, or those of the publisher, the editors and the reviewers. Any product that may be evaluated in this article, or claim that may be made by its manufacturer, is not guaranteed or endorsed by the publisher.
